# A role for jasmonates in the release of dormancy by cold stratification in wheat

**DOI:** 10.1093/jxb/erw172

**Published:** 2016-05-02

**Authors:** Qian Xu, Thy T. Truong, Jose M. Barrero, John V. Jacobsen, Charles H. Hocart, Frank Gubler

**Affiliations:** ^1^Shandong Agricultural University, College of Agronomy, Taian, Shandong, China; ^2^CSIRO Agriculture, Canberra ACT 2601, Australia; ^3^Mass Spectrometry Facility, Research School of Biology, Australian National University, Canberra ACT 2601, Australia

**Keywords:** Abscisic acid, acetylsalicylic acid, cold, dormancy, jasmonate, stratification, wheat.

## Abstract

Hydration at low temperatures, commonly referred to as cold stratification, is widely used for releasing dormancy and triggering germination in a wide range of species including wheat. However, the molecular mechanism that underlies its effect on germination has largely remained unknown. Our previous studies showed that methyl-jasmonate, a derivative of jasmonic acid (JA), promotes dormancy release in wheat. In this study, we found that cold-stimulated germination of dormant grains correlated with a transient increase in JA content and expression of JA biosynthesis genes in the dormant embryos after transfer to 20 ^o^C. The induction of JA production was dependent on the extent of cold imbibition and precedes germination. Blocking JA biosynthesis with acetylsalicylic acid (ASA) inhibited the cold-stimulated germination in a dose-dependent manner. In addition, we have explored the relationship between JA and abscisic acid (ABA), a well-known dormancy promoter, in cold regulation of dormancy. We found an inverse relationship between JA and ABA content in dormant wheat embryos following stratification. ABA content decreased rapidly in response to stratification, and the decrease was reversed by addition of ASA. Our results indicate that the action of JA on cold-stratified grains is mediated by suppression of two key ABA biosynthesis genes, *TaNCED1* and *TaNCED2*.

## Highlight

Dormancy release by cold stratification depends on jasmonate biosynthesis in wheat grains

## Introduction

Timing of germination is one of the most critical adaptation strategies used by plants to ensure reproductive success. In wild plant species, dormancy has a key role in ensuring survival of a population by blocking seed germination until conditions become favourable for germination and seedling establishment (Bewley, 1997). In addition to genetic determinants, environmental signals experienced during seed maturation and following dispersal strongly influence the timing of dormancy loss. Unlike many wild plant species, cultivated crops such as wheat (*Triticum aestivum* L.) display weak grain dormancy at maturity due to selective breeding against dormancy for uniform and vigorous germination. As a result, modern wheat varieties exhibit increased susceptibility to pre-harvest sprouting (PHS) following cool and moist conditions in the field, which result in serious loss of grain yield and quality (Gubler *et al.*, 2005; Rodríguez *et al.*, 2015). Thus, research aimed at understanding environmental and genetic control of dormancy will assist in developing new strategies for the elimination of PHS worldwide in domesticated crops.

A variety of environmental signals including temperature, light quality, photoperiod, and nitrate have been shown to influence cereal dormancy (Rodríguez *et al.*, 2015), with temperature widely recognized as the major signal (Harrington, 1923; Sawhney and Naylor, 1979; Buraas and Skinnes, 1985; Reddy *et al.*, 1985; Nyachiro *et al.*, 2002; Gu *et al.*, 2006). Interestingly, temperature effects on grain dormancy are very much dependent on the developmental stage. In general, temperature during grain development strongly influences dormancy, with cooler conditions producing more dormant grains (Barrero *et al.*, 2015). Exposure to higher temperatures for brief or extended periods during grain maturation has been shown to reduce dormancy (Gualano and Benech-Arnold, 2009).

There is also a dramatic change in the low temperature responses once grains are imbibed. Imbibition at 4 ^o^C, commonly referred to as cold stratification, is widely used to break dormancy (Bewley and Black, 1994). It is considered that cold stratification mimics the cold and moist conditions experience by cereals in soil during autumn and winter, during which seed dormancy is lost in order to trigger germination prior to spring. In wheat, stratification for 48h at 4 ^o^C in the dark is sufficient to induce germination even in strongly dormant cultivars (Mares, 1984). It is important to note that prematurely harvested grains as early as 18 d post-anthesis are capable of responding to stratification, although the effectiveness of the cold stratification increases with maturity (Gosling *et al.*, 1981). Even though the effect of cold is well known, the hormonal and molecular mechanisms by which cold stratification regulate cereal dormancy remain unknown.

Plant hormones play a key role in dormancy regulation through synergistic and antagonistic interactions. In cereals and other plants, abscisic acid (ABA) is considered to be the primary mediator in dormancy induction and maintenance (Finkelstein *et al.*, 2008; Rodríguez *et al.*, 2015; Shu *et al.*, 2016). Genes encoding ABA metabolism enzymes appear to be major targets for environmental signals that promote dormancy, such as hypoxia, high temperature, and blue light (BL) (Rodríguez *et al.*, 2015). Furthermore, dormancy release by after-ripening is mediated by a decrease in ABA content in imbibed wheat and barley grains as a result of co-ordinated promotion of ABA catabolism and repression of ABA biosynthesis genes (Millar *et al.*, 2006; Jacobsen *et al.*, 2013).

ABA action is regulated in part by crosstalk with other hormones such as gibberellins (GAs) and their associated signalling networks. Applications of GA, an antagonist of ABA, can be effective in breaking dormancy in cereals (Jacobsen *et al.*, 2002; Tuttle *et al.*, 2015). In Arabidopsis, GA is essential for germination, but this has not yet been demonstrated for cereals (Jacobsen *et al.*, 2002; Peng and Harberd, 2002; Ogawa *et al.*, 2003; Gubler *et al.*, 2005; Finkelstein *et al.*, 2008; Hauvermale *et al.*, 2015). A recent study has also demonstrated that jasmonates can act antagonistically to ABA by promoting dormancy release in wheat dormant grains (Jacobsen *et al.*, 2013). Applications of methyl-jasmonate (MeJA) inhibited the expression of the ABA biosynthesis gene, *Ta9-cis-EPOXYCAROTENOID DIOXYGENASE1* (*TaNCED1*), resulting in a decrease in ABA content in imbibed embryos prior to germination. However, in Arabidopsis, jasmonic acid (JA) and its precursor 12-*cis*-oxophytodienoic acid (OPDA) inhibited seed germination (Dave *et al.*, 2011), indicating that the role of JAs in dormancy varies according to the species. Other hormones such as ethylene, brassinosteroid, and auxin also play roles in germination of Arabidopsis, but as yet there is no information on whether they mediate dormancy processes in cereals (Steber and McCourt, 2001; Xue *et al.*, 2009; X, Liu *et al.*, 2013; Corbineau *et al.*, 2014).

Our current understanding of the role of hormones in cold stratification of seeds is very limited. In non-dormant Arabidopsis seeds, bioactive GA_4_ content increased in response to low temperature during imbibition, due—at least in part—to increased expression of a key GA biosynthesis gene (Yamauchi *et al.*, 2004). Mutant analysis revealed that the *AtGA3ox1* gene is required for cold-stimulated seed germination. However, this work has not been extended to test if this is also the case in imbibed dormant seeds. In wheat, changes in ABA sensitivity have been observed in response to stratification (Mares, 1984; Noda *et al.*, 1994), but it is not clear whether this is due to changes in ABA metabolism or signalling. Recently, a cold-induced increase in JA content was demonstrated in Arabidopsis seedlings exposed to cold. In this study, some genes of the JA biosynthesis pathway such as lipoxygenase (*LOX*), allene oxide synthase (*AOS*), and allene oxide cyclase (*AOC*) were induced by cold (Hu *et al.*, 2013). A cold-induced increase in JA has also been reported in rice roots (Moons *et al.*, 1997). Although we have previously demonstrated that MeJA and JA promote dormancy release in wheat (Jacobsen *et al.*, 2013) and that the JA biosynthesis pathway was up-regulated in the coleorhiza of non-dormant barley embryos compared with dormant grains (Barrero *et al.*, 2009), there was no evidence linking JA with cold responses in grains.

In this study we have undertaken hormonal and molecular analyses to investigate the role of JAs and other hormones in stratification of dormant wheat grains. Applying optimized reversed phase liquid chromatography with electrospray ionization tandem mass spectrometry (LC-ESI-MS/MS), we found that JA and jasmonyl-isoleucine (JA-Ile) contents increased rapidly in embryos of dormant grains following 48h stratification compared with grains that were incubated at 20 ^o^C. Molecular analysis revealed that the increase in JA and JA-Ile correlated with expression of cold-inducible JA biosynthesis genes. Furthermore, using a jasmonate biosynthesis inhibitor, we showed that jasmonate synthesis is required for cold-induced germination in wheat. The results also indicated that the increase in JA triggers a decrease in ABA content in the embryos of cold-stratified grains. The results are in agreement with our earlier study which showed that JA is a repressor of ABA biosynthesis (Jacobsen *et al.*, 2013), and they provide the first evidence that JAs play a central role in the release of seed dormancy by cold stratification.

## Materials and methods

### Plant materials

Two spring wheats cultivars (*Triticum aestivum* L. cv. Sunstate and cv. AC Barrie) were grown in a phytotron with the temperature set at 17 °C/9 °C day/night cycle as previously described (Gubler *et al.*, 2008). Sunstate is an Australian white-grained wheat and AC Barrie is a Canadian red-grained wheat. Heads were harvested at maturity, dried at 37 °C for 1 d, and grains were threshed by hand (to avoid grain damage and to remove the husk) and then stored at –20 °C to retain dormancy or after-ripened at 37 °C for 4 weeks.

### Chemicals

HPLC-grade acetone, diethyl ether, and methanol; citric acid, acetylsalicylic acid (ASA), ABA, and MeJA were from Sigma-Aldrich Co. JA, *cis*-OPDA, [^2^H_5_]OPDA, *N*-[(–)-jasmonoyl]-(l)-isoleucine, dihydrojasmonate (DHJA), and [^2^H_6_]ABA were from OlChemIm Ltd. *N*-[(±)–jasmonoyl]-(l)-isoleucine was from Paul E. Staswick.

### Germination assays

Germination was performed in Petri dishes each with 20 grains on one Whatman filter paper (No. 598) and 5ml of distilled water. Plates were sealed and incubated at 4 ^o^C or 20 ^o^C either under continuous 20 µmol m^−2^ s^−1^ BL-emitting diodes or wrapped in two layers of aluminium foil to block any light (dark treatment). Germinated grains were scored following emergence of coleorhiza (Barrero *et al.*, 2009). The germination index (GI) was calculated over 7 d imbibition as described in Walker-Simmonds (1987).

As in some experiments, grains were imbibed with ASA, MeJA, ABA, and OPDA. ASA was diluted with water to a 30mM stock solution; MeJA was prepared as a 2000 µM stock solution as previously described (Jacobsen *et al.*, 2013); and ABA was stored as a 30 µM stock and diluted prior to use.

### JA, JA-Ile, and ABA analysis

The extraction method (Dave *et al.*, 2011) was adapted to quantify the contents of JA, JA-Ile, and ABA in wheat embryos as follow.

With the aid of a microscope, biological replicates (*n*=4, each replicate weighing ~80mg) of 40 embryos were dissected from dry or imbibed wheat grains, weighed, and immediately frozen on dry ice. Samples were stored at –80 ^o^C until extraction. Subsequently, a 5mm stainless steel bead was placed in each tube to lyse the frozen embryos mechanically using a tissue lyser (Qiagen) for 15s at a speed of 30 cycles s^–1^. Then samples were spiked with 20 µl of the stock internal standard mixture (1 µg ml^−1^ dihydrojasmonic acid, [^2^H_5_]OPDA, and [^2^H_6_]ABA) and extracted with 1.8ml of acetone/50mM citric acid (70:30, v/v) for 3h at 4 ^o^C on an orbital shaker. Tubes were moved to a fumehood, uncapped, and left in darkness overnight for the acetone to evaporate. The remaining aqueous phase was extracted with diethyl ether (3×500 µl) and the extracts were combined in a 2ml glass vial and dried prior to reconstitution with 50 µl of methanol. Samples were filtered through a 0.45 µm GHP membrane (hydrophilic polypropylene) NanoSep MF centrifuge tube before LC/MS analysis.

Samples and standards (7 μl) were injected onto an Agilent Zorbax Eclipse 1.8 μm XDB-C18 2.1×50mm column. Solvent A consisted of 0.1% aqueous formic acid and solvent B, methanol with 0.1% formic acid. The plant hormones were eluted with a linear gradient from 10% to 50% solvent B over 8min, 50% to 70% solvent B from 8min to 12min (then held at 70% from 12min to 20min) at a flow rate of 200 μl min^–1^. The column effluent was analysed by an Agilent 6 530 Accurate Mass LC/MS QTOF with an ESI Jetstream ion source interface. Optimized ESI negative ion polarity parameters were as follow: gas temperature 250 ºC, drying gas flow 9 litres min^–1^, nebulizer 25 psig [pounds per square inch (gauge)], sheath gas temperature 250 ºC and sheath gas flow 11 litres min^–1^, capillary voltage 2500V, nozzle voltage 500V, and fragmentor voltage 145V. The positive ion polarity parameters were: gas temperature 250 ºC, drying gas flow 7 litres min^–1^, nebulizer 35 psig, sheath gas temperature 325 ºC and sheath gas flow 11 litres min^–1^, capillary voltage 3500V, nozzle voltage 500V, and fragmentor voltage 125V. The QTOF was operated in the extended dynamic range mode and data were acquired using targeted MS/MS (see Supplementary Table S1 at *JXB* online) with an *m/z* 1.3 isolation window prior to collision-induced dissociation (CID; N_2_ collision gas supplied at 18 psi). Mass spectra were acquired at three spectra s^–1^ and MS/MS at three spectra s^–1^ over a range of *m/*z 50–1000). The *m*/*z* values were corrected against two reference ions {purine, [MH]^+^ *m*/*z* 121.050873, [M-H]^−^ *m*/*z* 119.036320 and hexakis(1H, 1H, 3H-tetra fluoropropoxy)phosphazine, [MH]^+^ *m/z* 922.009798, [M+HCO_2_]^−^ *m/z* 966.000725}. Data were acquired and analysed using the Agilent Technologies MassHunter software (ver. B.5.0).

The extraction protocol of Dave *et al.* (2011) was validated by assessing absolute extraction recovery and matrix effects (i.e. ion suppression or ionization enhancement) using embryo samples that were unfortified (*n*=3) and fortified (*n*=3) with a known amount (50ng) of each analyte, and recovery (%) was calculated. Calibration standards (0.01–5.0ng µl^–1^), quality control (QC) standards (0.1ng µl^–1^ and 1.0ng µl^–1^), and samples were analysed, in both optimized positive and negative ion modes, for *cis*-OPDA, JA, JA-Ile, and ABA. Limits of detection (LOD) were determined by a signal to noise ratio of 3:1 based on the 7 µl injection volume, and the lower limits of quantification (LLOQ) were determined by a signal to noise ratio of 5:1 (see Supplementary Table S1). Calibration and QC standards had all internal standards fixed at a concentration of 0.4ng µl^–1^. Matrix effects were negligible. Absolute quantification was based on analyte peak areas normalized against peak areas of the corresponding isotope-labelled internal standard as shown in Supplementary Table S1. Quantification of JA and JA-Ile [(+)-7-iso-JA-l-Ile and (–)-JA-l-Ile] was performed against the analogue (±)-9,10-dihydrojasmonic acid internal standard.

### GA analysis

Samples were frozen in liquid nitrogen and lyophilized. GA analysis was carried out at the Plant Biotechnology Institute of the National Research Council of Canada (http://www.nrc-cnrc.gc.ca/eng/solutions/advisory/plant_hormone.html) using LC-MS/MS (Chiwocha *et al.*, 2003; Zaharia *et al.*, 2005). Four biological replicates were analysed.

### Quantitative PCR analysis

For RNA extraction, 10 embryos for each sample were ground with a tissue lyser and the powder added to 5ml of hot RNA lysis buffer as described by Chang *et al.* (1993). Following purification, the RNA was treated with RNase-free DNase. A 2 µg aliquot of total RNA was reverse transcribed in a 20 µl reaction. cDNA samples were diluted and used in 10 µl PCRs as described in Jacobsen *et al.* (2013). The reactions were run on a 7900HT fast real-time PCR system (Applied Biosystems). The expression of Ta*ACTIN* (CJ961169) (Ji *et al.*, 2011) was used as an internal control to normalize gene expression. For the analysis, a linear standard curve was generated using a series of dilutions for each PCR product; the levels of the transcript in all unknown samples were determined according to the standard curve. The sequence of primers and gene accession number are listed in Supplementary Table S2. Two biological repeats were performed with similar results. Data from one of the replicates are shown.

### Accession numbers

Full-length or EST sequence data in this work can be found in the EMBL/GenBank database under the following accession numbers: wheat *TaAOS1* (AY196004), *TaAOS2* (BT009396), *TaAOC1* (KF573524), *TaAOC2* (BJ241555), *TaAOC3* (CK163974), *TaNCED1* (CD884104), and *TaNCED2* (CA731387).

## Results

### Endogenous JA and JA-Ile increase in response to cold stratification

**Fig. 1.**
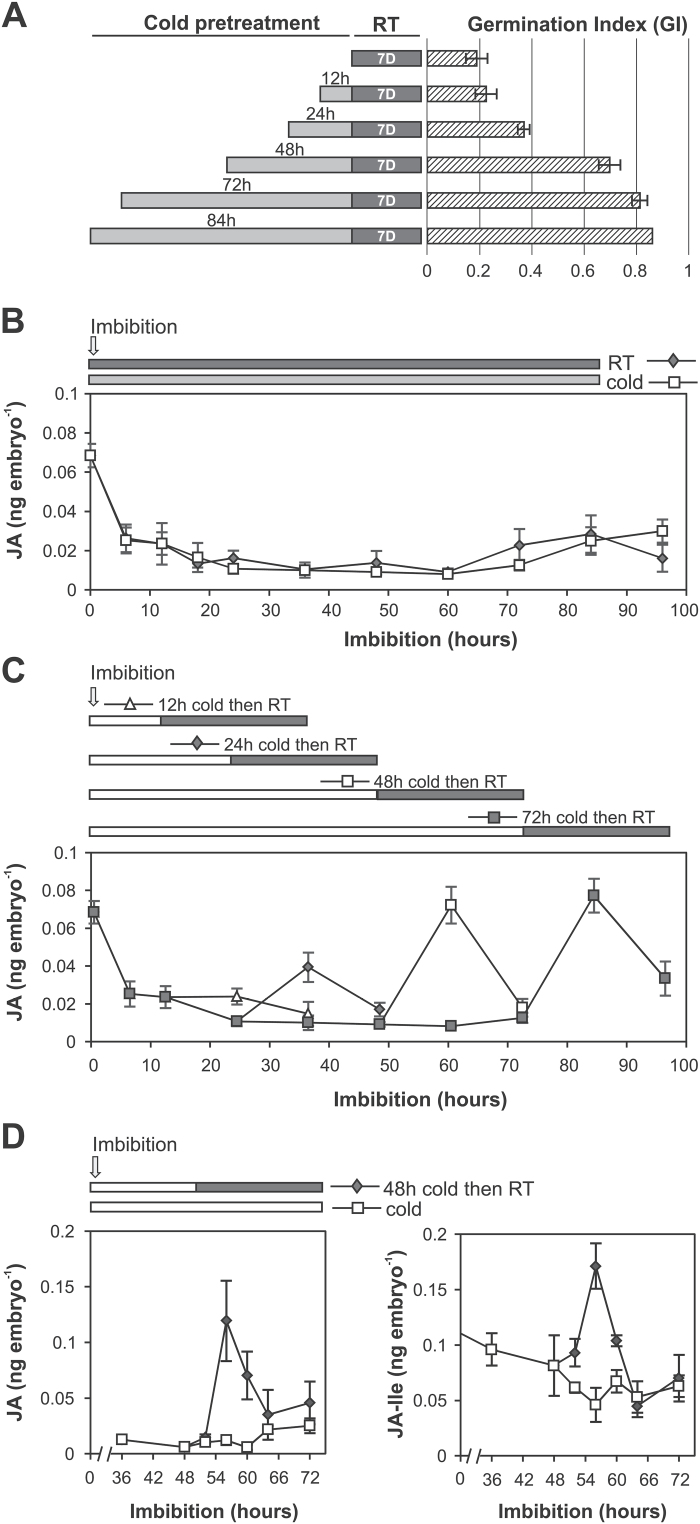
JA and JA-Ile increase in embryos of imbibed wheat grains in response to cold. (A) The effect of stratification on the germination of dormant grain. The GI was calculated over 7 d imbibition at 20 ^o^C after different periods of cold treatment (0–84h). (B) JA content in embryos isolated from Sunstate wheat grains imbibed for 96h continuously in the cold or at 20 ^o^C. (C) Changes in JA content in embryos isolated from wheat grains that have been stratified for different times (12, 24, 48, and 72h) prior to imbibition at 20 ^o^C. (D) Changes in JA and JA-Ile content in embryos of wheat grains in response to 48h stratification. Grains were imbibed at 4 ^o^C for 48h and then transferred to room temperature (RT) or kept at 4 ^o^C. Values are means ±SE (*n*=4), and each replicate was obtained from 20 grains (A) and 40 embryos (B–D).

To characterize the responsiveness to stratification, dormant wheat grains (cv. Sunstate) were imbibed under BL (conditions in which dormancy is manifested to a greater extent) in the cold (4 ^o^C) for periods of up to 84h prior to transfer to room temperature (20 ^o^C) for 7 d (Jacobsen *et al.*, 2013). Germination was assessed over the 7 d period and the effect of stratification time on the GI was measured (Fig. 1A; Supplementary Fig. S1). GI increased with duration of cold stratification until 48h when dormancy was almost completely lost. Similarly, longer stratification for 72h and 84h resulted in only further small increases in GI. The observed response to stratification is similar to that previously published for other wheat cultivars (Mares, 1984; Noda *et al.*, 1994).

To assess JA changes in response to continuous cold treatment, we compared JA content in embryos of dormant grains imbibed continuously in the cold and at room temperature over a 96h period (Fig. 1B). The JA content was highest in dry embryos and it decreased during the first 6h of imbibition both in the cold and at room temperature, and thereafter remained low in both treatments up to 96h imbibition.

We then measured JA production in wheat embryos following stratification. JA was quantified in dormant wheat grains that were imbibed in the cold for different times (12, 24, 48, and 72h) and transferred to room temperature for 24h (Fig. 1C). JA content increased in wheat embryos that had been stratified for longer than 12h. The increase in JA content in the stratified grains correlated with the loss of dormancy, with close to maximal effect observed in grains that were stratified for 48h or longer prior to transfer to 20 ^o^C (Fig. 1A, C).

To understand further the induction of JA production, we repeated the analysis of 48h stratified grains with more time points to better define changes in the content of JA and its associated metabolite, JA-Ile, following the cold treatment. As shown in Fig. 1D, JA content in embryos increased (~10-fold) between 4h and 8h after transfer to 20 ^o^C, and thereafter the content decreased rapidly back to levels similar to those found in wheat grains imbibed continuously in the cold. JA-Ile, one of the JA bioactive forms, also increased (~2-fold) in a pattern similar to JA, reaching its maximum between 4h and 8h after transfer to room temperature (Fig. 1D).

We also examined the effect of dark on germination and JA content in cold-treated grains. In contrast to BL, imbibition of cv. Sunstate grains under dark conditions strongly promoted germination (Supplementary Fig. S2A). Analysis of JA content in grains imbibed in the dark revealed that following 48h stratification, JA content increased rapidly 12h after transfer to room temperature (Supplementary Fig. S2B). We were also interested in determining if the cold-induced increase in JA could be observed in a more highly dormant wheat variety. Wheat cv. AC Barrie was found to be dormant even when imbibed under dark conditions (Supplementary Fig. S2A); however, germination of cv. AC Barrie grains was strongly promoted by 48h imbibition at 4 ^o^C in the dark prior to transfer to room temperature (Supplementary Fig. S2A). As shown in Supplementary Fig. S2B, changes in JA content were similar to those found in cv. Sunstate grains, indicating that the cold-induced increase in JA content is not cultivar specific.

### Effect of stratification on the jasmonate biosynthesis pathway

**Fig. 2.**
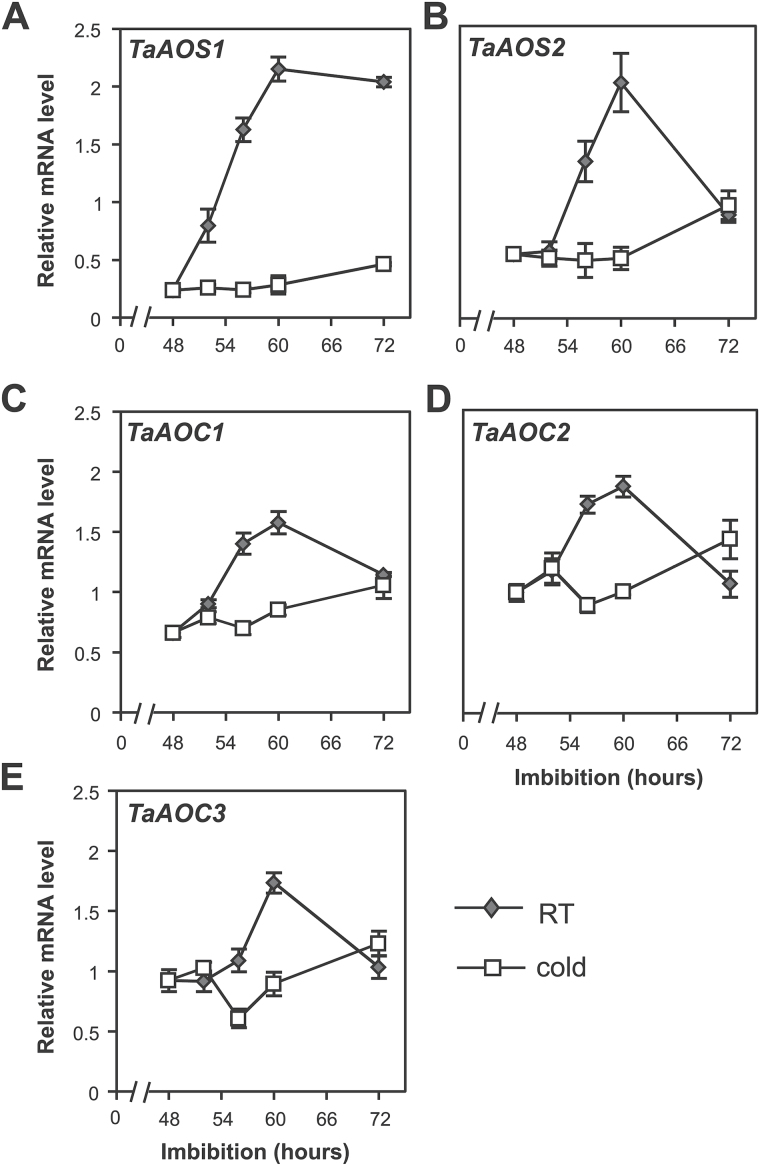
Expression of JA biosynthesis genes is induced by 48h stratification in wheat grains. Relative expression of *TaAOS1* (A), *TaAOS2* (B), *TaAOC1* (C), *TaAOC2* (D), and *TaAOC3* (E) in embryos of wheat grains following 48h stratification. Grains were imbibed at 4 ^o^C for 48h and then either transferred to room temperature (RT) or kept at 4 ^o^C up to 24h (52, 56, 60, and 72h). Values are means ±SE (*n*=4), and each replicate was obtained from 10 embryos.

To understand further the effect of stratification on JA production, we examined the responses of several genes in the jasmonate biosynthesis pathway to cold. In Arabidopsis, *AOS* and *AOC* genes are considered to encode key enzymes in jasmonate biosynthesis and to be regulated by cold (Hu *et al.*, 2013). On the basis of published sequences of *AOS* and *AOC* genes in wheat (Wu *et al.*, 2004; Liu *et al.*, 2011; A. Liu *et al.*, 2013; Zhao *et al.*, 2014), we selected *TaAOS1*, *TaAOS2*, *TaAOC1*, *TaAOC2*, and *TaAOC3* genes and monitored their expression after stratification. In response to stratification, the expression of *TaAOS* (Fig. 2A, B) and *TaAOC* (Fig. 2C, E) genes was up-regulated within 8h after transfer to room temperature. These data suggest that the increase in JA content is due, at least in part, to up-regulation of the expression of *TaAOS* and *TaAOC* genes in wheat embryos (Fig. 2).

### Inhibition of jasmonate biosynthesis blocks the stratification response

**Fig. 3.**
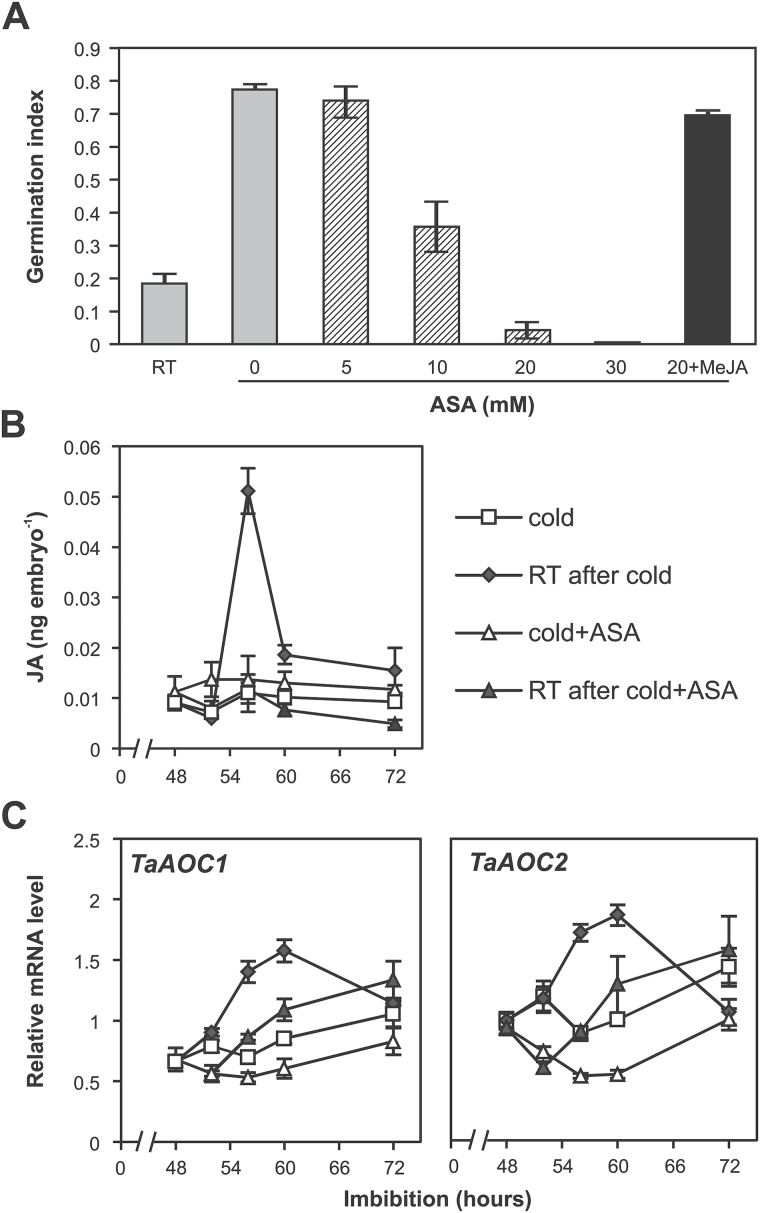
JA biosynthesis is required for cold-induced germination in dormant wheat. (A) Effect of ASA and MeJA on the GI of wheat following a 48h cold treatment. Different concentrations of ASA (5, 10, 20, and 30mM) and 100 µM MeJA were added from the beginning of cold imbibition. Values are means ±SE (*n*=4), and each replicate was obtained from 20 grains. (B) Effect of ASA on JA content in embryos of imbibed wheat grains following a 48h cold treatment. Grains were imbibed for 2 d with or without ASA in the cold before being transferred to room temperature (RT). For comparison, grains were kept in the cold after the 48h cold treatment. Embryos were isolated for JA quantification after 48 (end of cold treatment), 52, 56, 60, and 72h imbibition. Values are means ±SE (*n*=4), and each replicate was obtained from 40 embryos. (C) Effect of ASA on expression of *TaAOC1* and *TaAOC2* in embryos of imbibed wheat grains in response to a 48h cold treatment. Grains were imbibed for 2 d with or without ASA in the cold before being transferred to RT. For comparison, grains were kept in the cold after the 48h cold treatment. Embryos were isolated for gene expression test after 48 (end of cold treatment), 52, 56, 60, and 72h imbibition. Values are means ±SE (*n*=4), and each replicate was obtained from 10 embryos.

ASA has been shown to block JA synthesis in tomato and flax leaves (Pena-Cortés *et al.*, 1993; Harms *et al.*, 1998). We have used ASA to test whether the increase in JA content is required for the stratification response in wheat. Figure 3A shows that different concentrations of ASA (5, 10, 20, and 30mM) suppressed the cold effect on germination in a dose-dependent manner. In addition, we tried to rescue the effect of ASA by adding 100 µM MeJA. The addition of MeJA reversed the inhibitory effect of ASA, which suggests that JA biosynthesis is essential for the stratification response and the effect is not due to non-specific effects of ASA. We also confirmed that ASA blocked the cold-induced increase in JA content (Fig. 3B). Addition of 20mM ASA blocked the cold-induced peak of JA production, which provided a direct link between the increase in JA and the cold effect on germination. ASA temporally blocked the cold-induced expression of *TaAOC1* and *TaAOC2*, indicating that they may play an essential role in mediating the effect of cold on JA content (Fig. 3C). We did not detect significant down-regulation of *TaAOS1*, *TaAOS2*, and *TaAOC3* genes (Supplementary Fig. S3) in response to ASA.

### Stratification induces a decrease in ABA content

**Fig. 4.**
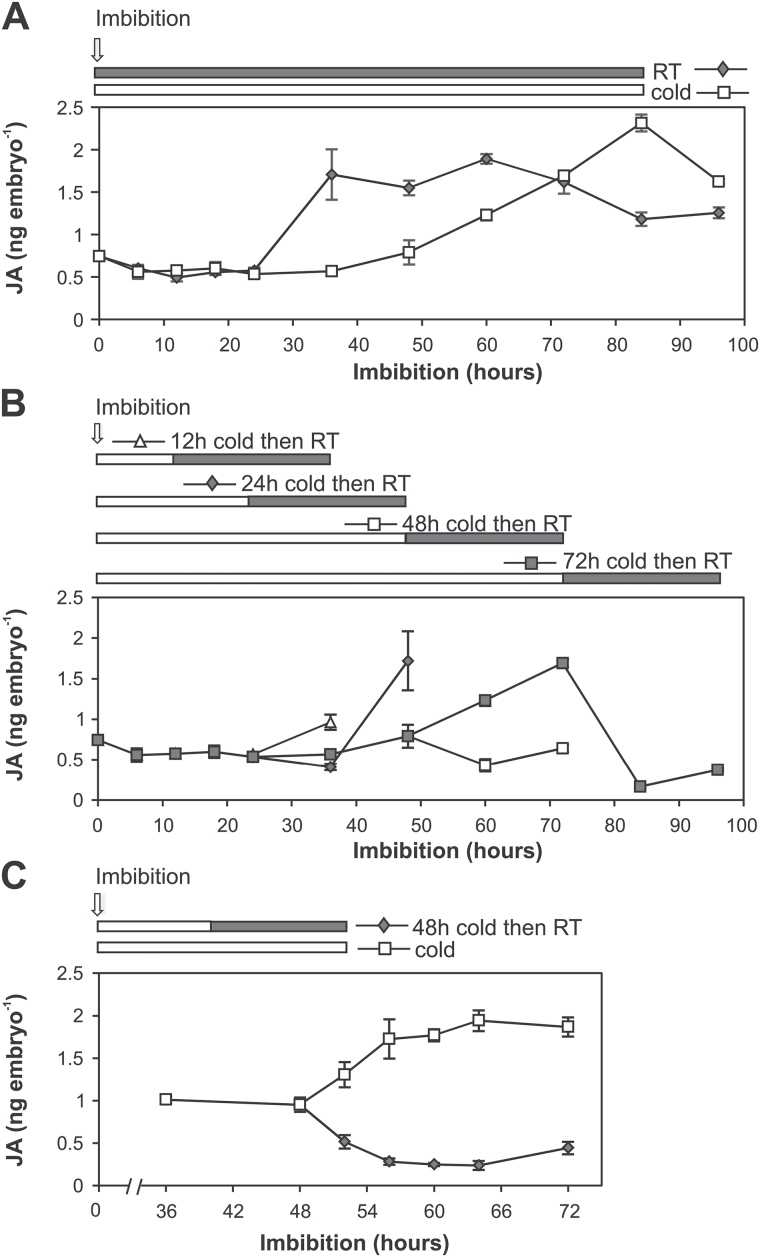
Changes in ABA content in embryos of imbibed wheat grain in response to stratification. (A) Time course of endogenous ABA content in embryos imbibed at 4 ^o^C (cold) and 20 ^o^C [room temperature (RT)). (B) Changes in ABA content in embryos of grains imbibed for different periods of time in the cold (12, 24, 48, and 72h) prior to transfer to RT. (C) More detailed time course of changes in ABA content in embryos of wheat grains in response to 48h stratification. Dormant grains were imbibed at 4 ^o^C for 48h before transfer to RT. For comparison, grains were also kept in the cold after 48h. Values are means ±SE (*n*=4), and each replicate was obtained from 40 embryos.

In order to understand the effect of cold on other hormones, we also investigated the role of ABA and GA in the stratification response. In contrast to changes observed with JA, ABA content increased in embryos of wheat grains that were imbibed continuously at either 4 ^o^C or 20 ^o^C but the increase was delayed in grains imbibed at the lower temperature (Fig. 4A). As demonstrated previously, ABA content of embryos of dormant grains increased after 24h imbibition at room temperature and peaked by 48h (Jacobsen *et al.*, 2013). ABA content in cold-imbibed grains increased after 48h imbibition and then continued to rise until 84h. When we examined the effect of stratification time on ABA content, it was observed that stratification times longer than 24h resulted in a decrease in ABA content in the 24h period after transfer to room temperature. Shorter stratification times (12h and 24h) resulted in increased ABA content in the embryo (Fig. 4B) similar to what happened in the room temperature control.

**Fig. 5.**
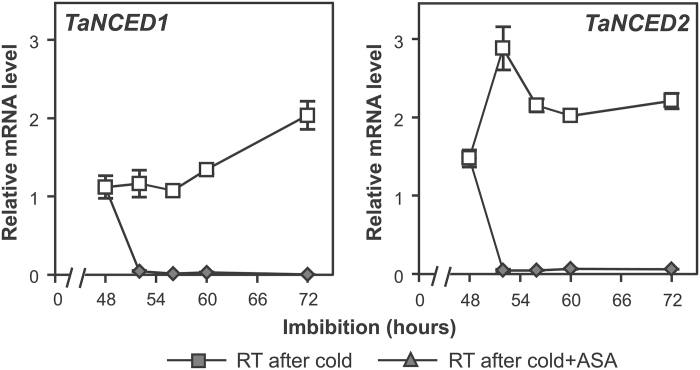
Expression of ABA biosynthesis genes, *TaNCED1* and *TaNCED2*, is repressed in embryos of wheat grains following stratification. Dormant grains were imbibed at 4 ^o^C for 48h before transfer to room temperature (RT).The grains were imbibed in the cold for 48h prior to transfer to RT. For comparison, grains were also kept in the cold after the 48h cold treatment. Values are means ±SE (*n*=4), and each replicate was obtained from 10 embryos.

To examine this more closely, changes in ABA content were monitored every 4h following a 48h stratification treatment. As shown in Fig. 4C, ABA content started to decrease within 4h of transfer from 4 ^o^C to 20 ^o^C, and, by 8h, it reached the minimum. The decrease in ABA content correlated with the decrease in expression of *TaNCED1* and *TaNCED2*, both key ABA biosynthesis genes expressed in wheat embryos (Jacobsen *et al.*, 2013) (Fig. 5). The decrease in ABA levels was also observed in grains that were imbibed in the dark (Supplementary Fig. S2C).

**Fig. 6.**
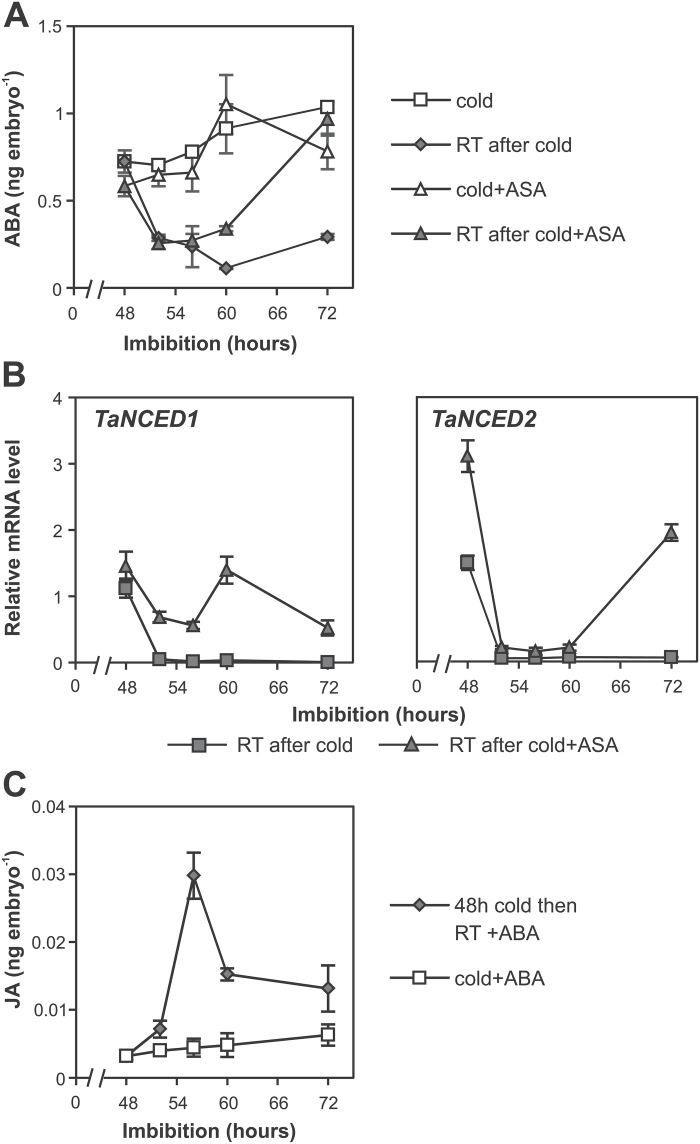
JA regulates ABA content in embryos of wheat grains in response to stratification. (A) ASA promotes an increase in ABA content in embryos of imbibed wheat grains at room temperature (RT) following a 48h cold treatment. Grains were imbibed in the cold for 48h and then either transferred to RT or kept in the cold. (B) ASA-induced changes in relative *TaNCED1* and *TaNCED2* transcript abundance in embryos following a 48h stratification. Grains were imbibed in the cold for 48h and then either transferred to RT or kept in the cold. Two biological repeats were performed with similar results. Data from one of the replicates are shown. (C) Addition of 100 µM ABA to the imbibition media does not block cold-induced increases in JA content in embryos of stratified wheat grains. Grains were imbibed in the cold for 48h and then either transferred to RT or kept in the cold. Values are means ±SE (*n*=4), and each replicate was obtained from 40 embryos (A and C) and 10 embryos (B).

Since it had been previously shown that MeJA represses *TaNCED1* expression and ABA content in wheat embryos (Jacobsen *et al.*, 2013), we tested whether the decrease in ABA content following the 48h stratification is directly linked to the increase in JA content. Changes in ABA content were monitored in 48h stratified wheat grains imbibed in the presence of ASA. As shown in Fig. 6A, ABA content in embryos was higher in the ASA-treated grains imbibed at room temperature for 24h following the stratification compared with no ASA treatment. The higher ABA content correlated with the increase in expression of *TaNCED1* and *TaNCED2* in ASA-treated grains (Fig. 6B). To assess further the relationship between the JA and ABA during the cold-stimulated germination process, JA content was examined after addition of a high concentration of ABA (100 µM). The induction peak of JA still occurred in the presence of ABA (Fig. 6C). These data led to the conclusion that JA acts upstream of ABA during wheat germination of stratified grains.

Examination of changes in GA and GA metabolites identified significant increases in the content of some GA precursors (GA_53_ and GA_44_) and of a GA catabolite (GA_8_) in embryos of grains imbibed at room temperature following 48h stratification (Supplementary Fig. S4). However, no bioactive GAs (GA_1_, GA_3_, or GA_4_) were detected in this experiment (Supplementary Table S3). To test whether GA biosynthesis is required for cold-induced promotion of germination in dormant grains, grains were imbibed in paclobutrazol, a well known inhibitor of GA biosynthesis, including in wheat grains (Lenton *et al.*, 1994). Addition of 10 μM paclobutrazol to the imbibition medium failed to block the effect of cold stratification on germination yet it reduced the maximum elongation rate of leaf 1 compared with control treatments (Supplementary Fig. S5A–C). It is also of interest to note that addition of GA alone failed to promote germination of stratified and non-stratified dormant grains.

### JAs have no detectable role in dormancy release by after-ripening and darkness

Following the demonstration that JA plays a role in cold-induced dormancy release, we looked to see whether the role could be extended to other dormancy release mechanisms. To determine whether JA had a role in dormancy release by dry after-ripening, JA content was monitored over a 60 d after-ripening period at 37 ^o^C during which grains progressively lost dormancy (Supplementary Fig. S6A). As shown in Supplementary Fig. S6B, little or no change in JA, JA-Ile, and ABA content in embryos of dry grains was observed over the 60 d period. We also determined whether there were any differences in JA and JA-Ile content during imbibition of dormant and after-ripened grain (Supplementary Fig. S7B, C). Apart from a difference in JA content at 36h imbibition between dormant and after-ripened grains, there was no evidence that JAs had a role in the after-ripening response. Imbibition in the dark also promoted germination of dormant grains (Supplementary Fig. S7A), but again no difference in JA content was observed between dormant grains imbibed in the dark or in the light (Supplementary Fig. S7C, C).

## Discussion

JA and its metabolites are ubiquitous signalling compounds known to regulate multiple processes including defence responses (Browse, 2009), root elongation (Staswick *et al.*, 1992; Pauwels *et al.*, 2010), freezing responses (Hu *et al.*, 2013), male fertility (McConn and Browse, 1996; Cheng *et al.*, 2009), senescence (Ueda and Kato, 1980; Shan *et al.*, 2011), anthocyanin accumulation (Franceschi and Grimes, 1991; Shan *et al.*, 2009), and flowering time (Diallo *et al.*, 2014). In the present work, we have identified several lines of evidence that point to a new role for JA in dormancy release by cold stratification. First, we have found that JA biosynthesis in wheat embryos is induced following cold stratification of dormant grains and that the increase in JA content correlates with the effect on germination. The effect of cold stratification on JA accumulation was observed in wheat cultivars of different dormancy and was independent of light conditions used during imbibition. Importantly, the increase in JA content in cold-stratified grains, which happened at 4–8h (Fig. 1D; Supplementary Fig. S2B) after transfer to room temperature, precedes the emergence of the coleorhiza (the earliest visual indication of grain germination after 8h; Supplementary Fig. S8), thus suggesting a causal link between the two events. We explored the possibility of JA being produced as a consequence of germination; however, that scenario was excluded as our results showed that there is no consistent change in JA and JA-Ile content in embryos of non-dormant grains that are undergoing germination (Supplementary Fig. S7A, B). Secondly, experiments with a JA biosynthesis inhibitor showed that JA production is sufficient and necessary for dormancy release by stratification. This is consistent with our previous results which showed that application of MeJA and JA reduces dormancy of wheat grains (Jacobsen *et al.*, 2013). Thirdly, our study provided evidence that the action of JA on dormancy release is at least in part mediated by reduced ABA content in embryos caused by reduced expression of ABA biosynthesis genes.

Cold-induced increases in JA content have been previously reported in plants. A small increase in JA-Ile (<25%) and no change in JA content have been reported in embryos of wheat grains imbibed for 18h at 30 ^o^C following a 24h stratification treatment compared with embryos from non-stratified grains (Tuttle *et al.*, 2015). It is important to note that the 24h stratification used in the study only had a small effect on the GI of wheat grains imbibed in water. However, the authors reported that the short stratification time impacted on the sensitivity of the grains to GA and ABA. In general, our results support this result but also extend it to demonstrate that the transient increases in JA and JA-Ile occur much earlier, with the maxima achieved 8h after the stratification. Our data show a 10-fold increase in JA content and a 2-fold increase in JA-Ile content in embryos within the first 8h following transfer to 20 ^o^C after stratification. Similar differences are found when the JA content in embryos 8h after stratification are compared with grains that are incubated continuously at 20 ^o^C (non-stratified grains) or 4 ^o^C (compare Fig. 1B and D).

Cold-induced increases in MeJA have also been reported in wheat seedlings (Diallo *et al.*, 2014). MeJA accumulates in winter wheat during vernalization and then decreases rapidly once the plants are transferred to inductive flowering conditions. The decrease in MeJA precedes the rise in expression of *TaVRN1* and *TaFT1*, indicating that MeJA may suppress expression of flowering time genes during vernalization and thus delaying flowering until the plants are exposed to warmer temperatures (Diallo *et al.*, 2014). It is now clear that vernalization and stratification in wheat are both mediated at least in part by the accumulation of JAs. It is intriguing to note that in both instances accumulation in JAs precedes activation of growth processes both in the embryonic axis and in the vegetative apex.

The role of JAs may not only vary depending on the developmental stage of various tissue types and between seeds from different species. The JA response to stratification in wheat grains appears to be fundamentally different from that in Arabidopsis, since it only occurs after the cold treatment, once the grains are transferred to room temperature. In Arabidopsis seedlings, JA accumulated rapidly within 1.5h of transfer from 22 ^o^C to 4 ^o^C, triggering the expression of the INDUCER OF CBF EXPRESSION-C-REPEAT BINDING FACTOR transcriptional pathway (Hu *et al.*, 2013). This response appeared to be related to cold stress responses, and the increase in JA enhanced the seedling freezing tolerance.

Our data indicate that in wheat dormancy, the role of JAs is restricted to cold stratification and not other dormancy release mechanisms such as after-ripening or darkness. We found that JA and JA-Ile contents were very similar in embryos from dormant and after-ripened grain imbibed over 24h at 20 ^o^C, with content declining 3- to 4-fold over this period. This is in agreement with an earlier study of wheat grain dormancy which found that JA and JA-Ile content declined in whole dormant and after-ripened grains over 24h hydration at room temperature (A. Liu *et al.*, 2013). Similarly, we found little difference between dormant grains that were imbibed in the dark or BL even though the grains had different germination outcomes (Supplementary Fig. S7A, B).

We have previously shown that wheat grains have different responses to MeJA depending on the dormancy status. Application of MeJA to dormant grain strongly promoted dormancy release, but in after-ripened grain the application of MeJA inhibited the growth of the embryo (Jacobsen *et al.*, 2013). The dependence on the dormancy status in determining the role of JA may also explain why there are reports of JAs promoting or repressing germination in a number of other plant species (Daletskaya and Sembdner, 1989; Berestetsky *et al.*, 1991; Ranjan and Lewak, 1992; Jarvis *et al.*, 1997; Krock *et al.*, 2002; Yildiz *et al.*, 2007, 2008; Preston *et al.*, 2009). This dual role of JA could explain why the cold-induced JA production is transitory; otherwise it would affect embryo growth once dormancy is released.

The role of JAs in seeds hase been previously studied in Arabidopsis, but their functions seem different from those in wheat grains. For example, large differences in JA and JA-Ile content have been reported in dry seeds from Arabidopsis ecotypes which differ in their level of dormancy, the content higher being in non-dormant than in dormant ecotypes (Preston *et al.*, 2009). However, so far, there is no functional evidence supporting a role for that correlation. Moreover, the role of JA and OPDA has also been examined in non-dormant Arabidopsis seeds. In that work, using single and double mutants, it was established that OPDA has an inhibitory effect on germination by increasing ABA sensitivity, and that JA and JA-Ile had no effect (Dave *et al.*, 2011). In wheat, the application of OPDA has small effects promoting germination (Supplementary Fig. S9), and the content of this molecule was below the level of detection in our stratification assays. Taken together, these results suggest that the role of JA, JA-Ile, and OPDA in dormancy and germination could be different between dicot and monocot species, although more genetic work is needed to confirm this conclusion.

In Arabidopsis seedlings, cold rapidly up-regulates expression of a number of key genes in the JA biosynthesis pathway including *LOX1–LOX3*, *AOS*, *AOC1–AOC4*, and *JAR1* (Hu *et al.*, 2013). We found in wheat that the expression of *TaAOS1-2* and *TaAOC1-3* increased rapidly only after the cold stratification treatment and that this correlated with the rapid rise in JA content. ASA blocked the cold-induced increase in JA content and germination, possibly in part by temporally suppressing the cold-induced expression of *TaAOC1* and *TaAOC2* genes. Furthermore this evidence confirms the contribution of both *TaAOC* genes to the cold-induced transient increase in JA. In flax leaves, ASA has been shown to block JA production via inhibition of *AOS* expression (Harms *et al.*, 1998), indicating that ASA is able to alter the expression of different JA biosynthesis genes in different plants. It is important to note that recent work has shown that salicylic acid inhibition of JA biosynthesis is a consequence of feedback inhibition caused by repression of JA signalling (Leon-Reyes *et al.*, 2010; van der Does et al., 2013).

The role of ABA as a principal dormancy promoter and germination inhibitor has been extensively reported (Finkelstein *et al.*, 2008). However, little is known about crosstalk between JA and ABA signalling pathways in cereals. ABA and JA show a synergistic relationship in response to several abiotic and biotic stresses such as wounding, salinity, or pathogen attack (Wang *et al.*, 2001; Forcat *et al.*, 2008; Wang *et al.*, 2012). In contrast, an antagonistic interaction between the signalling pathways for those hormones has been recorded for other stresses (Moons *et al.*, 1997; Anderson *et al.*, 2004; Nakata *et al.*, 2013), thus indicating that the nature of ABA and JA crosstalk is complex and stress dependent. In relation to wheat grains, we have identified an inverse relationship between JA and ABA content in response to 48h stratification, and we have asked if the two responses are inter-related. Our results show that the decrease in ABA content precedes the increase in JA content and that ABA content remains low after the transient spike in JA content. Our results are consistent with a recent report which found that a 24h cold stratification treatment of wheat grains was associated with a small decrease in ABA and a small increase in JA-Ile content (Tuttle *et al.*, 2015). We explored the possibility that ABA might suppress JA synthesis in wheat embryos by determining whether ABA could suppress JA production. Addition of 100 μM ABA to wheat grains undergoing cold stratification failed to block the production of a transient spike in JA, indicating that the increase in JA and the initial rapid decrease in ABA content in response to cold stratification are independent events. However, we were able to show that JA may have a key role in maintenance of low ABA content following the rapid initial decrease in response to cold. By blocking JA production in cold-stratified grains with ASA, we were able to show that ABA content rapidly increased to levels similar to those found before stratification. We were also able to show that the increase in ABA content in ASA-treated grains can be attributed to a promotion of expression of the key ABA biosynthesis genes, *TaNCED1* and *TaNCED2*. This was consistent with our previous finding that MeJA promotion of dormancy release in wheat acts via inhibition of ABA biosynthesis by suppressing *TaNCED1* expression in embryos of dormant grains (Jacobsen *et al.*, 2013). Therefore, the decline and maintenance of low ABA content in stratified grains is, at least in part, directly attributed to JA suppression of ABA synthesis. It has been shown that dormant wheat grains lose ABA sensitivity in response to cold (Noda *et al.*, 1994; Tuttle *et al.*, 2015), but it has yet to be determined if the change in ABA sensitivity is due to cold-induced changes in ABA signalling mechanisms. However, it is important to note that manipulation of expression of ABA metabolism genes can also lead to alterations in ABA sensitivity (Okamoto *et al.*, 2006). Thus, JA-induced changes in ABA content may be responsible, at least in part, for changes in ABA sensitivity.

Previous studies of seed germination have established a link between cold stratification and GA. In particular, increases in GA content during cold stratification have been observed in seeds of several plant species (West, 1970; Rudnioki *et al.*, 1972; Yamauchi *et al.*, 2004; Chen *et al.*, 2008). This evidence, along with the demonstration that GA application is often effective in breaking dormancy, has led to the proposal that cold-induced increases in GA content are responsible for increased germination (Yamauchi *et al.*, 2004). However, in agreement with a previous study (Tuttle *et al.*, 2015), we were not able to detect any bioactive GAs in our cold-stratified grains, indicating that the content of these GAs is very low and thus likely not to have a role. The demonstration that the GA biosynthesis inhibitor paclobutrazol did not inhibit cold stratification promotion of germination provides evidence that GA biosynthesis is not critical for the response in wheat. In contrast, we were able to detect changes in some GA precursors and catabolites, indicating that GA metabolism is active in cold-treated grains but its role remains unknown.

**Fig. 7.**
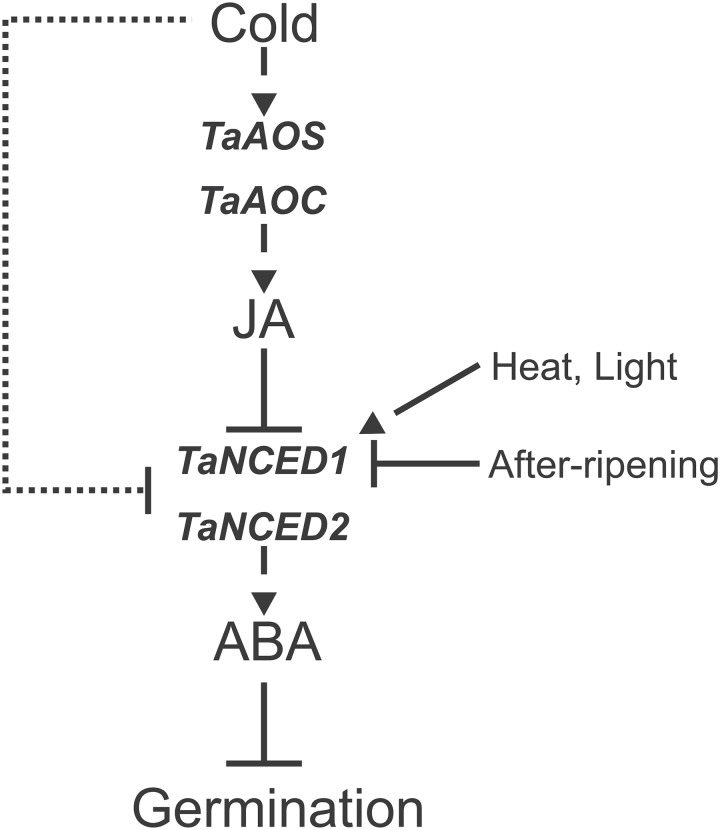
Model summarizing the role of JA and ABA in dormancy release by cold stratification in wheat. Stratification stimulates rapid changes in two hormones known to regulate dormancy. First, there is a decrease in ABA content, independent of JA (dotted line), which is driven by the repression of *TaNCED1* and *TaNCED2*. Secondly, there is a cold-induced increase in JA content that also decreases ABA by repressing the *NCED* genes. The increase in JA is driven by the cold-induced expression of *TaAOS* and *TaAOC*. Blockage of JA biosynthesis using ASA results in high ABA content; thus, the maintenance of low ABA after stratification is dependent on JA production. In cereals, *NCED1* is a common key target of environmental factors (cold, heat, light, and after-ripening) that affect germination.

In conclusion, our study in wheat leads us to propose a model showing that JA and ABA have opposing roles in the regulation of dormancy release by stratification (Fig. 7). ABA repression of germination acts through two signalling pathways, one independent of JA (dotted line) and one dependent on JA signalling mechanisms. We showed that the cold-induced increase in JA content is necessary for dormancy release and that its action is mediated, at least in part, by repression of ABA biosynthesis genes, *TaNCED1* and *TaNCED2*. *NCED1* in wheat and barley has been previously shown to be regulated by a number of other environmental conditions such as high temperature stress (Leymarie *et al.*, 2008), BL (Jacobsen *et al.*, 2013), and after-ripening (Gubler *et al.*, 2008). In addition, we have now shown that both *TaNCED1* and *TaNCED2* are down-regulated in response to cold in a JA-independent pathway (dotted line). Further investigations are now needed to determine how cold stratification promotes JA biosynthesis and at the same time inhibits ABA biosynthesis.

## Supplementary data

Supplementary data are available at *JXB* online.

Figure S1. The effect of stratification on the germination of dormant grain.

Figure S2. Changes in JA and ABA content in embryos of imbibed Sunstate and AC Barrie grain in response to stratification in darkness.

Figure S3. Effect of ASA on expression of *TaAOC1*, *TaAOC2*, and *TaAOS3* in embryos of imbibed wheat grains in response to 48h stratification.

Figure S5. Effect of paclobutrazol and gibberellic acid on GI and leaf 1 elongation rate.

Figure S4. Changes in GA content in embryos of Sunstate wheat grains in response to 48h stratification.

Figure S6. JA and ABA contents in dry embryos during after-ripening.

Figure S7. Time course of JA content in dormant and after-ripened embryos in BL and darkness.

Figure S8. Time course of germination of Sunstate wheat grains in response to 48h stratification.

Figure S9. Effect of ODPA on germination on dormant and after-ripened wheat grains.

Table S1. Validation results for extraction and LC-ESI-MS/MS analytical methodologies for the detection and absolute quantification of plant hormones in wheat embryos.

Table S2. Primer sequences used for qRT–PCR.

Table S3. Changes in GA content in embryos of Sunstate wheat grains in response to 48h stratification in BL.

Supplementary Data
