# Supplementary material for: A role for jasmonates in the release of dormancy by cold stratification in wheat
Source: J Exp Bot. 2016 May 2;67(11):3497–508. doi: 10.1093/jxb/erw172 (PMC4892733; doi:10.1093/jxb/erw172)
Supplement: Supplementary Data [file supp_67_11_3497__index.html]

A role for jasmonates in the release of dormancy by cold stratification in wheat — A role for jasmonates in the release of dormancy by cold stratification in wheat — Supplementary Data 

# A role for jasmonates in the release of dormancy by cold stratification in wheat

## Supplementary Data

Data files

- supplementary\_figures\_S1\_S9\_tables\_S1\_S3.pdf - Supplementary Data
