# Supplementary material for: A role for jasmonates in the release of dormancy by cold stratification in wheat
Source: J Exp Bot. 2016 May 2;67(11):3497–508. doi: 10.1093/jxb/erw172 (PMC4892733; doi:10.1093/jxb/erw172)
Supplement: Supplementary Data [file supp_erw172_supplementary_figures_S1_S9_tables_S1_S3.pdf]

Supplemental Figure S1

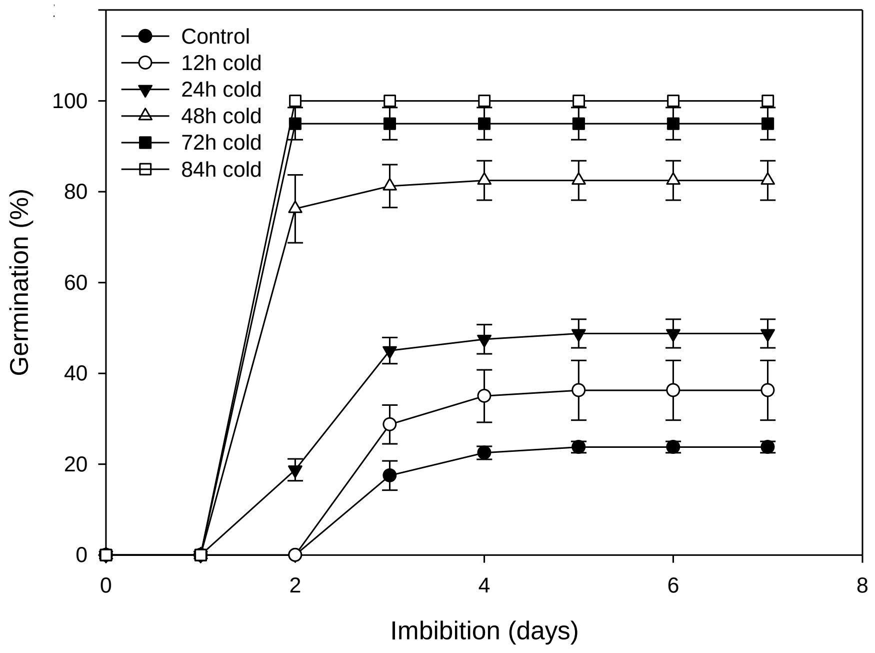

Supplemental Figure S1. The effect of stratification on the germination of dormant grain. The germination was measured over 7 days' imbibition at 20°C following different periods of cold treatment (0 to 84 h).

Supplemental Figure S2

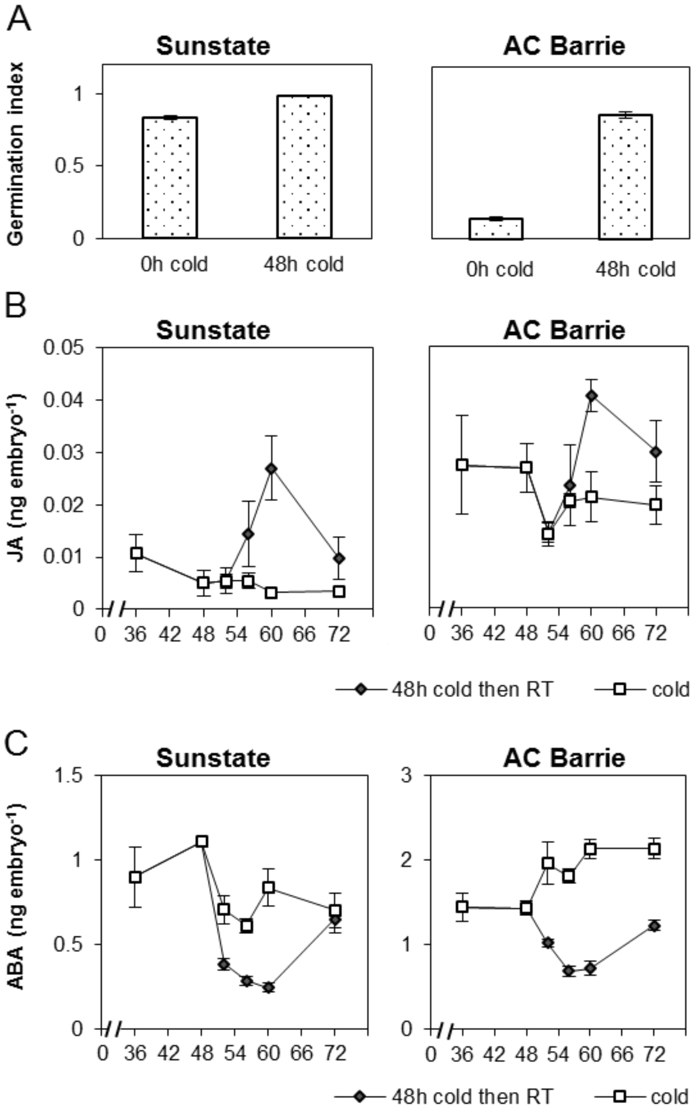

Supplemental Figure S2. Changes in JA and ABA content in embryos of imbibed wheat grains in response to stratification in darkness. (A) The effect of 48 h stratification on the germination of dormant cv. Sunstate and cv. AC Barrie grains in darkness. (B) Changes in JA content in embryos isolated from cv. Sunstate and cv. AC Barrie wheat grains in response to 48 h stratification in dark. Grains were imbibed at 4°C for 48 h in dark and then transferred to RT in dark or kept at 4°C in dark. (C) Changes in ABA content in embryos isolated from cv. Sunstate and cv. AC Barrie wheat grains in response to 48 h stratification in dark. Grains were imbibed at 4°C for 48 h in dark and then transferred to RT in dark or kept at 4°C in dark. Values are means ±SE (n=4) and each replicate was obtained from 20 grains (A) and 40 embryos (B-C).

Supplemental Figure S3

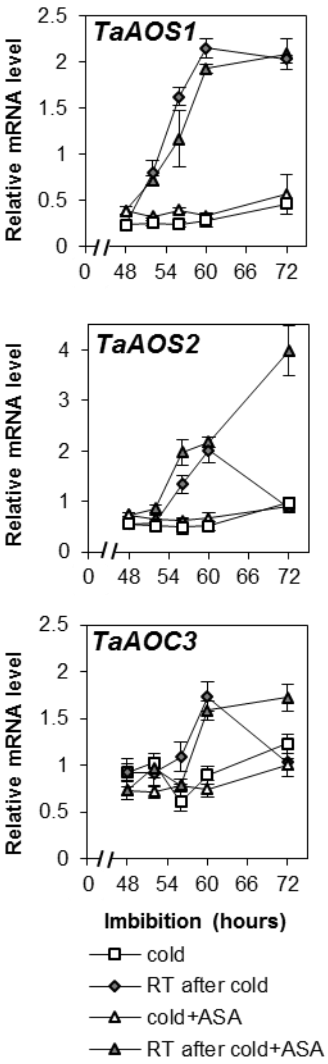

Supplemental Figure S3. Effect of ASA on expression of *TaAOC1, TaAOC2* and *TaAOS3* in embryos of imbibed wheat grains in response to 48 h stratification. Cv. Sunstate wheat grains were imbibed for 48 h with or without ASA in BL in the cold before being transferred to RT. For comparison, grains were kept in the cold after the 48 h cold treatment. Embryos were isolated for gene expression analysis after 48 h (end of cold treatment), 52 h, 56 h, 60 h and 72 h imbibition. Values are means ±SE (n=4) and each replicate was obtained from 10 embryos.

Supplemental Figure S4

A

GA53 → **GA44** → **GA19** → GA20 (→GA29) →
GA1(bioactive form) →**GA8**

B

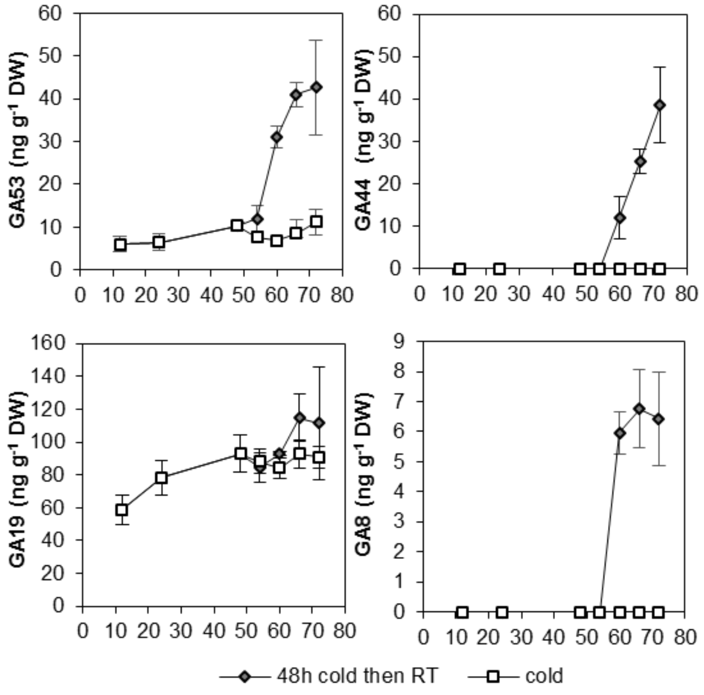

Supplemental Figure S4. Changes in GA content in embryos of Sunstate wheat grains in response to 48 h stratification. (A) Partial GA metabolic pathway in plants. (B) GA contents in imbibed wheat embryos in response to cold..$GA_{53,}$ GA_{44} and GA_8 increase in embryos of cv. Sunstate wheat grains in response to 48 h stratification. Grains were imbibed at 4^oC for 48 h under BL and then transferred to RT under BL or kept at 4^oC under BL. Values are means ±SE (n=4) and each replicate was obtained from 40 embryos. DW, dry weight.

Supplemental Figure S5

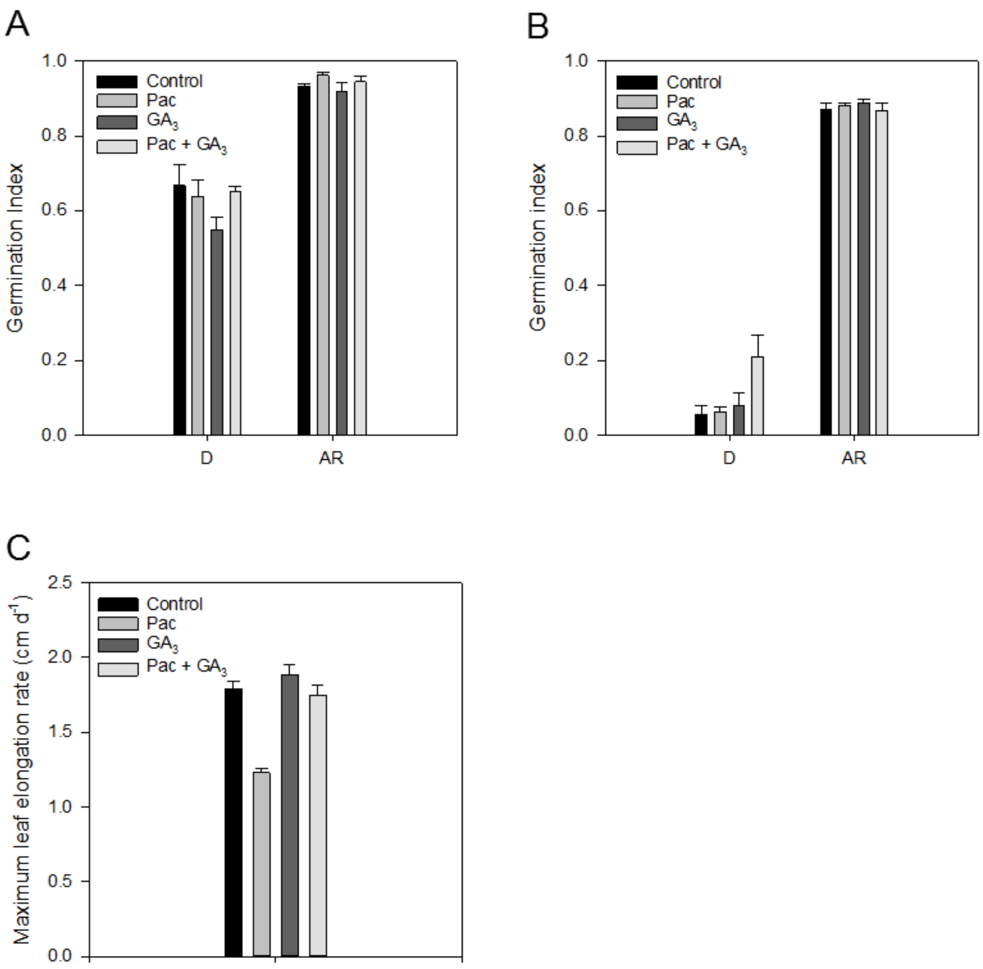

Supplemental Figure S5. Effect of paclobutrazol and gibberellic acid on germination index and leaf 1 elongation rate. (A) The effect of paclobutrazol (Pac) and gibberellic acid (GA_3) on germination of dormant (D) and after-ripened (AR) grains following 48 h stratification. At the start of the stratification the grains were imbibed in control, 10 μM paclobutrazol, 50 μM GA_3 or 10 μM paclobutrazol and 50 μM GA_3. Paclobutrazol was made as a 10 mM stock in DMSO and diluted 1000 fold. Control and GA_3 treatments included addition of DMSO at the same concentration as the paclobutrazol treatments. Grains were imbibed at 4°C for 48 h under BL and then transferred to RT for 7 days under BL. Germination index of grains was calculated over the 7 days. Values are means ±SE (n=4) and each replicate was obtained from 20 grains. (B) The effect of paclobutrazol (Pac) and GA_3 on germination of D and AR grains imbibed at RT. The grains were imbibed in control, 10 μM paclobutrazol, 50 μM GA_3 or 10 μM paclobutrazol and 50 μM GA_3. Paclobutrazol was made as a 10 mM stock in DMSO and diluted 1000 fold. Control and GA_3 treatments included addition of DMSO at the same concentration as the paclobutrazol treatments. Grains were imbibed for 7 days under BL. Germination index of grains was calculated over the 7 days. Values are means ±SE (n=4) and each replicate was obtained from 20 grains. (C) Effect of paclobutrazol and GA_3 on leaf 1 elongation rate. The maximal leaf elongation rate for leaf 1 was determined as described by Chandler and Robertson (1999) except that Sunstate wheat grains were not stratified prior to germination. After-ripened grains were placed in paper envelopes moistened with various treatments: control, 10 μm paclobutrazol (Pac), 50 μM GA_3, or 10 μM paclobutrazol and 50 μM GA_3. The envelopes were incubated under constant white light for 7 days. Lengths of leaf 1 from 18-25 seedlings were measured daily from day 3 to day 7. Error bars represent standard error.

Chandler PM, Robertson M. 1999. Gibberellin dose–response curves and the characterization of dwarf mutants of barley. Plant Physiology 120, 623–632.

Supplemental Figure S6

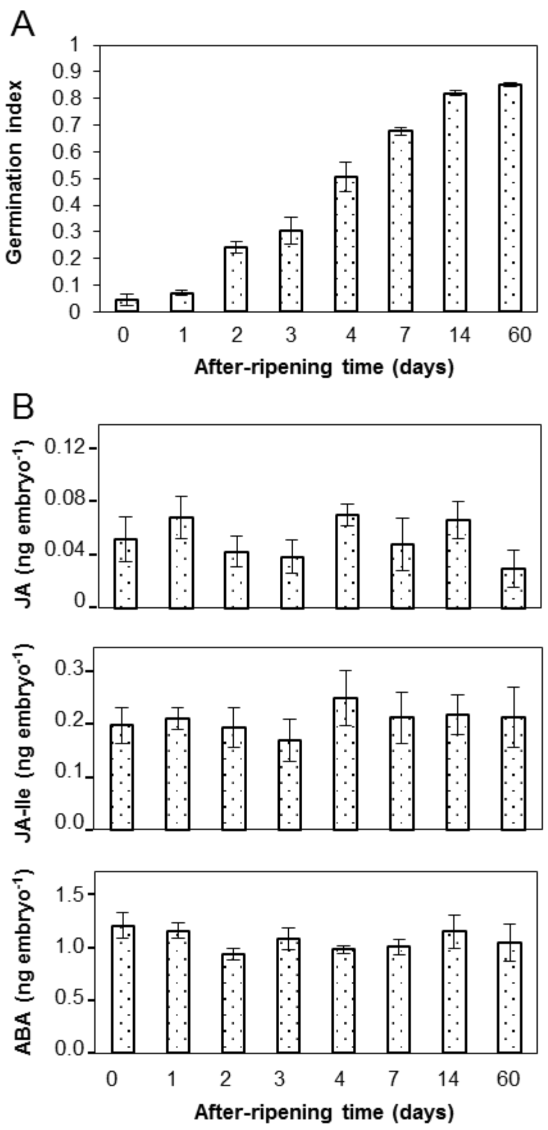

Supplemental Figure S6. JAs and ABA contents in dry embryos during after-ripening. (A) After-ripening increases the GI of wheat.GI was calculated over 7 days of imbibition in BL at 20° C following different periods of after-ripening in 37ºC . Values are means ±SE (n=4) and each replicate was obtained from 20 grains. (B) JA, JA-Ile and ABA contents in embryo of dry seeds during after-ripening. Embryos were isolated for hormone quantification after different times of after-ripening. Values are means ± SE (n=4) and each replicate was obtained from 25 embryos.

Supplemental Figure S7

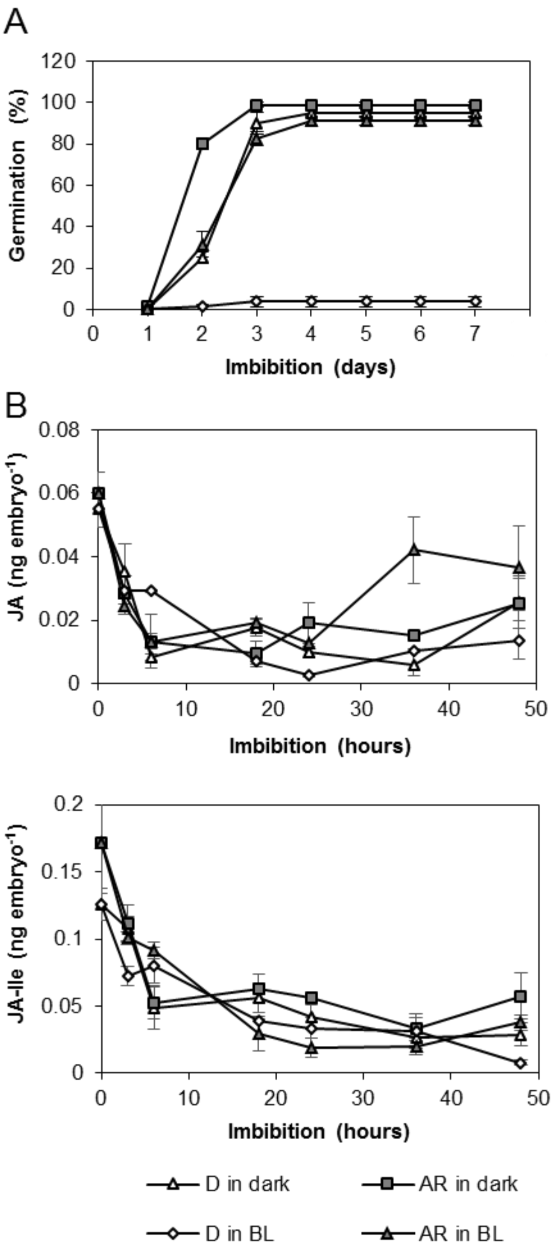

Supplemental Figure S7. Time course of JAs content in dormant and after-ripened embryos in BL and darkness. (A) The effect of BL and darkness on germination of dormant and after-ripened wheat. Germination rate of dormant and after-ripened was calculated for 7 days in BL and dark. Values are means ± SE (n=4) and each replicate was obtained from 20 grains. (B) JA and JA-Ile contents in dormant and after-ripened embryos in BL and in dark during imbibition. Embryos were isolated for hormone quantification after different times of after-ripening. Values are means ± SE (n=4) and each replicate was obtained from 40 embryos.

Supplemental Figure S8

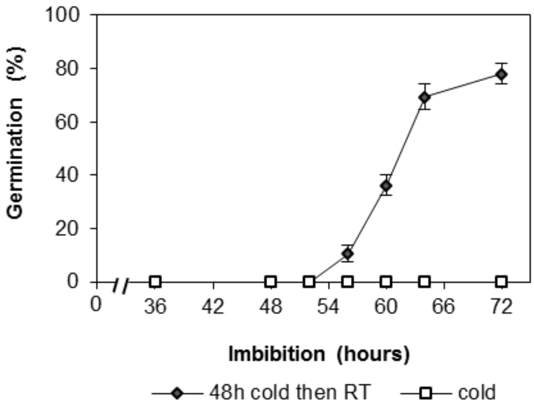

Supplemental Figure S8. Time course of germination of Sunstate wheat grains in response to 48 h stratification**.** Germination rate of dormant wheat grains in response to 48 h stratification. Grains were imbibed at 4°C for 48 h under BL and then transferred to RT under BL or kept at 4°C under BL. Values are means ±SE (n=4) and each replicate was obtained from 20 grains.

Supplemental Figure S9

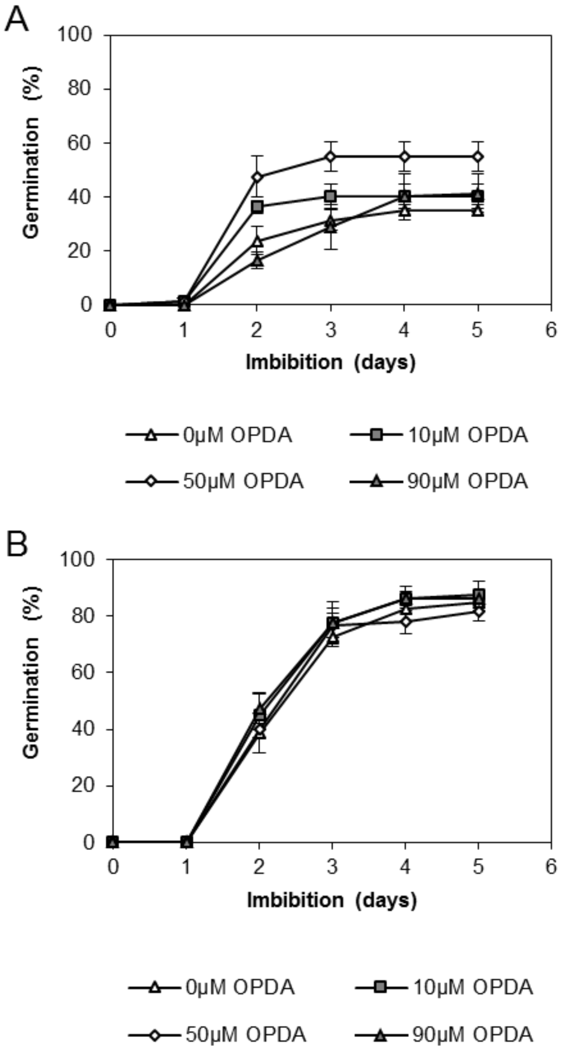

Supplemental Figure S9. Effect of ODPA on germination on dormant and after-ripened wheat grains**.** (A) OPDA promotes the germination of dormant wheat. The germination rate of dormant was scored for 5 days in BL (10μmol·m^{-2}·sec^{-1}) and the different concentrations of OPDA. Values are means ±SE (n=4) and each replicate was obtained from 20 embryos. (B) OPDA has no effect on germination of after-ripened wheat. The germination rate of after-ripened grains was scored for 5 days in BL (10µmol·m^{-2}·sec^{-1}) and different concentration of OPDA. Values are means ±SE (n=4) and each replicate was obtained from 20 embryos.

Supplementary Table S1. Validation results for extraction and LC-ESI-MS/MS analytical methodologies for the detection and absolute quantification of plant hormones in wheat embryos

Analyte	Molecular formula	MW (g mol^{-1})	Parent ion (*m/z*)	CE (eV)	t_R (min)	Base Peak Product ion (*m/z*)	LOD (pg/µg)	LLOQ (pg/µg)	Linear regression equation	Correlation coefficient (R^2)	Recovery efficiency (%)	Optimised ESI-QTOF Ion Polarity
(±)-Jasmonic acid (JA)	$C_{12}H_{18}O_3$	210.27	209.1163	12	14.0602 ± 0.2721	59.0144	0.2647	0.4111	y = 0.8689x + 0.0457	0.9902	90±0.06	Negative
N-[(-)-Jasmonoyl]-(L)-isoleucine ((-)-Ja-L-Ile))	$C_{18}H_{29}NO_4$	323.21	322.2027	20	16.612 ± 0.2363	130.0878	0.2186	0.3643	y = 1.2109x + 0.0061	0.9915	99±0.04	Negative
(±)-9,10-DihydroJasmonic acid (DHJA)	$C_{12}H_{20}O_3$	212.28	211.1349	12	15.3150 ± 0.2332	59.0163	0.0523	0.0871				Negative
(±)-*cis,trans*-Abscisic acid (ABA)	$C_{15}H_{20}O_4$	264.32	263.1297	10	12.8344 ± 0.2842	153.0935	0.1801	0.3002	y = 2.4495x + 0.0018	0.9974	85±0.03	Negative
[$^2H_6$](+)-*cis,trans*-Abscisic acid (D-ABA)	d_6-$C_{15}H_{14}O_4$	270.35	269.1647	10	12.7952 ± 0.2843	159.1294	0.4109	0.6848				Negative
Cis-12-oxo-phytodienoic acid (*cis*-OPDA)	$C_{18}H_{28}O_3$	292.42	293.2095	7	20.6835 ± 0.0281	275.1994	0.9518	1.5864	y = 419.9x + 2.0084	0.9974	N/A	Positive
[$^2H_5$]*cis*-12-Oxo-phytodienoic acid (D-OPDA)	$C_{18}H_{23}D_5O_3$	297.38	298.2252	15	20.6169 ± 0.0316	280.2160	2.5930	4.3216				Positive

Limits of detection (LOD = 3xS/N) and lower limits of quantification (LLOQ = 5xS/N) were calculated according to the calibration curves of individual analyte standards. Standards were run in both negative and positive ion polarities, and the respective ion mode with better detection sensitivity was selected for each analyte. Results for the extraction recovery (%) of each targeted analyte (n=3, means ± %CV); retention times (n=10; means ± SE); optimal collision energies to fragment the parent ions to generate their respective characteristic product ions; calibration curve linearity and correlation coefficients. The presence of internal standards (analogues and isotope-labeled) was necessary to achieve reliable accuracy and precision of the extraction and LC/MS methods.

Supplementary Table S2. Primer sequences used for qRT-PCR

Gene	Forward primer (5'-3')	Reverse primer (5'-3')	Accession
TaAOS1	TCTCATAGCAGCCGTCAATC	CACATACACAAGGGCACCAT	AY196004
TaAOS2	ATGGTGCCCTTGTGTATGTG	TACCGTACGCCAAGAGTGAG	BT009396
TaAOC1	CGTCTTCGAGGGCGTCTACG	GCAGGTCGGGGATGCCCTTGA	KF573524
TaAOC2	ATTCATTCAACACTGGTACAAGG	ATCTATTATTGCTCCTGCTAGTAG	BJ241555
TaAOC3	TCCTCGTAGGTGAGGTAGGG	CTACGAGGCCATCTACAGCA	CK163974
TaNCED1	CCCAGCACTAATCGATTCC	CCGCTAACTGTATCCATGC	CD884104
TaNCED2	GGAGATGGAAAGAGGAAGTCG	GAAGCAAGTGTGAGCTAAC	CA731387

Supplemental Table S3. Changes in GAs content in embryos of Sunstate wheat grains in response to 48 h stratification in BL.

			Gibberellins (ng/g DW)													
Sample information		weight (mg)	GA1	GA3	GA4	GA7	GA8	GA9	GA19	GA20	GA24	GA29	GA34	GA44	GA51	GA53
1A	12h in 4°C	52	n.d.	<3.8	<3.8	n.d.	<3.8	n.d.	50	<3.8	<3.8	<4.4	<3.8	<3.8	n.d.	4
1B	12h in 4°C	48	n.d.	n.d.	n.d.	n.d.	<4.1	n.d.	55	<4.1	n.d.	n.d.	n.d.	<4.1	n.d.	7
1C	12h in 4°C	52	n.d.	<3.9	<3.9	<3.9	<3.9	n.d.	59	<3.9	n.d.	n.d.	<3.9	<3.9	n.d.	4
1D	12h in 4°C	52	n.d.	n.d.	<3.8	<3.8	<3.8	n.d.	71	<3.8	<3.8	n.d.	<3.8	<3.8	n.d.	8
2A	24h in 4°C	48	n.d.	n.d.	n.d.	<4.2	<4.2	n.d.	72	<4.2	<4.2	<5.8	<4.2	n.d.	n.d.	5
2B	24h in 4°C	44	<4.5	n.d.	<5.4	<4.5	<4.5	n.d.	91	<4.5	<4.5	n.d.	<4.5	n.d.	n.d.	6
2C	24h in 4°C	52	n.d.	<3.8	<3.8	<3.8	<3.8	n.d.	83	<3.8	<3.8	n.d.	<3.8	<3.8	n.d.	9
2D	24h in 4°C	49	n.d.	<4	n.d.	<4	<4	n.d.	68	<4	<4	n.d.	n.d.	<4	n.d.	5
3A	48h in 4°C	39	n.d.	<5.1	n.d.	n.d.	<5.1	n.d.	92	<5.1	<5.1	n.d.	<5.1	<5.1	n.d.	10
3B	48h in 4°C	37	n.d.	<5.5	n.d.	n.d.	<5.5	n.d.	101	<5.5	n.d.	n.d.	<5.5	<5.5	n.d.	10
3C	48h in 4°C	47	n.d.	n.d.	n.d.	n.d.	<4.3	n.d.	77	<4.3	n.d.	n.d.	<4.3	<4.3	<4.3	9
3D	48h in 4°C	39	n.d.	n.d.	n.d.	<5.2	<5.2	n.d.	101	<5.2	n.d.	<6.7	n.d.	n.d.	n.d.	11
4A	54h in 4°C	31	n.d.	n.d.	n.d.	n.d.	n.d.	n.d.	99	n.d.	n.d.	n.d.	n.d.	<6.4	n.d.	7
4B	54h in 4°C	33	n.d.	<6.1	<6.1	n.d.	n.d.	n.d.	86	<6.1	n.d.	n.d.	n.d.	n.d.	n.d.	8
4C	54h in 4°C	37	n.d.	n.d.	<5.4	n.d.	<5.7	n.d.	81	<5.4	<5.4	n.d.	<5.4	n.d.	n.d.	7
4D	54h in 4°C	41	n.d.	<4.8	n.d.	n.d.	n.d.	n.d.	88	<4.8	n.d.	n.d.	n.d.	<4.8	n.d.	8
5A	60h in 4°C	49	n.d.	<4.1	n.d.	<4.1	n.d.	n.d.	78	<4.1	<4.1	n.d.	<4.1	<4.1	n.d.	7
5B	60h in 4°C	35	n.d.	n.d.	<5.8	<5.8	<5.8	n.d.	93	<5.8	n.d.	<7.9	<5.8	<5.8	n.d.	8
5C	60h in 4°C	39	n.d.	n.d.	n.d.	n.d.	<5.1	n.d.	86	<5.1	n.d.	n.d.	<5.1	<5.1	n.d.	5
5D	60h in 4°C	44	n.d.	n.d.	<4.6	<4.6	<4.6	n.d.	80	<4.6	n.d.	n.d.	n.d.	<4.6	n.d.	7
6A	66h in 4°C	40	n.d.	n.d.	<5	n.d.	<5	n.d.	91	<5	n.d.	n.d.	<5	<6.6	<5	10
6B	66h in 4°C	45	n.d.	<4.5	<4.5	<4.5	<4.5	n.d.	87	<4.5	n.d.	n.d.	<4.5	<4.5	n.d.	9
6C	66h in 4°C	45	n.d.	n.d.	<4.4	n.d.	<4.6	n.d.	88	<4.4	<4.4	<6.7	<4.4	n.d.	n.d.	8
6D	66h in 4°C	38	n.d.	n.d.	n.d.	n.d.	<5.2	n.d.	105	<5.2	n.d.	n.d.	n.d.	<5.2	<5.2	8
7A	72h in 4°C	34	n.d.	<5.8	n.d.	n.d.	n.d.	n.d.	98	<5.8	<5.8	n.d.	n.d.	<5.8	n.d.	14
7B	72h in 4°C	36	n.d.	n.d.	n.d.	n.d.	<5.5	n.d.	87	<5.5	<5.5	<7.4	n.d.	<5.5	n.d.	8
7C	72h in 4°C	41	n.d.	<4.8	n.d.	n.d.	<4.8	n.d.	84	<4.8	<4.8	<10.7	n.d.	<4.8	n.d.	9
7D	72h in 4°C	36	n.d.	n.d.	<5.6	n.d.	n.d.	n.d.	94	<5.6	n.d.	n.d.	<5.6	<5.6	<5.8	14
8A	48h in 4°C+ 6h in 20°C	41	n.d.	n.d.	n.d.	n.d.	<4.9	n.d.	81	<5.3	n.d.	n.d.	<4.9	<4.9	n.d.	11
8B	48h in 4°C+ 6h in 20°C	51	n.d.	n.d.	n.d.	n.d.	<3.9	n.d.	75	<3.9	n.d.	n.d.	<3.9	<3.9	<3.9	9
8C	48h in 4°C+ 6h in 20°C	33	n.d.	n.d.	<6	n.d.	<6	n.d.	96	<6	n.d.	n.d.	<6	<6	n.d.	16
8D	48h in 4°C+ 6h in 20°C	38	n.d.	<5.3	n.d.	<5.3	<5.3	n.d.	86	<5.3	n.d.	n.d.	n.d.	<5.3	n.d.	12
9A	48h in 4°C+ 12h in 20°C	44	<4.6	n.d.	n.d.	<4.6	<4.6	n.d.	94	n.d.	<4.6	n.d.	<4.6	8	n.d.	28
9B	48h in 4°C+ 12h in 20°C	47	n.d.	n.d.	n.d.	<4.3	<4.3	n.d.	92	<4.3	n.d.	n.d.	n.d.	7	n.d.	30
9C	48h in 4°C+ 12h in 20°C	37	n.d.	<5.3	<5.3	<5.3	5	n.d.	94	<5.3	n.d.	n.d.	n.d.	15	<7.2	32
9D	48h in 4°C+ 12h in 20°C	37	n.d.	n.d.	n.d.	n.d.	6	n.d.	92	<5.5	n.d.	n.d.	n.d.	17	<5.5	34
10A	48h in 4°C+ 18h in 20°C	44	n.d.	n.d.	n.d.	n.d.	6	n.d.	103	<4.6	n.d.	n.d.	n.d.	24	n.d.	44
10B	48h in 4°C+ 18h in 20°C	45	n.d.	n.d.	n.d.	n.d.	7	n.d.	101	<4.5	n.d.	n.d.	n.d.	23	n.d.	42
10C	48h in 4°C+ 18h in 20°C	40	n.d.	n.d.	<5	<5	9	n.d.	128	<5	<5	n.d.	<5	25	<5	37
10D	48h in 4°C+ 18h in 20°C	48	n.d.	n.d.	<4.2	n.d.	6	n.d.	127	<4.2	n.d.	n.d.	n.d.	29	n.d.	41
11A	48h in 4°C+ 24h in 20°C	51	n.d.	n.d.	n.d.	n.d.	4	n.d.	66	<3.9	n.d.	n.d.	n.d.	31	n.d.	31
11B	48h in 4°C+ 24h in 20°C	40	n.d.	n.d.	n.d.	n.d.	7	n.d.	144	<5	n.d.	<5.1	<5	50	n.d.	58
11C	48h in 4°C+ 24h in 20°C	49	n.d.	<4.1	n.d.	<4.1	7	n.d.	105	n.d.	n.d.	n.d.	n.d.	41	n.d.	42
11D	48h in 4°C+ 24h in 20°C	50	n.d.	n.d.	n.d.	n.d.	8	n.d.	131	<4	n.d.	n.d.	<4	32	<4	40

Values greater than or equal to the limits of quantification (LOQ ≤ 8 ng/g DW) are reported in red. Values in blue indicate concentrations (ng/g DW) that were below the LOQs, but required reporting.

***n.d.* = not detected (values below LOQs of 3 ng/g DW). LODs = 3xS/N.**

LEGEND:

	A,B,C,D	**Gibberellins**		
	four replicates		GA1	Gibberellin 1
			GA3	Gibberellin 3
	1: 12h in 4°C in BL		GA4	Gibberellin 4
	2: 24h in 4°C in BL		GA7	Gibberellin 7
	3:48h in 4°C in BL		GA8	Gibberellin 8
	4:54h in 4°C in BL		GA9	Gibberellin 9
	5:60h in 4°C in BL		GA19	Gibberellin 19
	6:66h in 4°C in BL		GA20	Gibberellin 20
	7:72h in 4°C in BL		GA24	Gibberellin 24
	8:48h in 4°C in BL+ 6h in 20°C in BL		GA29	Gibberellin 29
	9:48h in 4°C in BL+ 12h in 20°C in BL		GA34	Gibberellin 34
	10:48h in 4°C in BL+ 18h in 20°C in BL		GA44	Gibberellin 44
	11:48h in 4°C in BL+ 24h in 20°C in BL		GA51	Gibberellin 51
			GA53	Gibberellin 53
